# The Association between Serum Hemoglobin and Renal Prognosis of IgA Nephropathy

**DOI:** 10.3390/jcm10020363

**Published:** 2021-01-19

**Authors:** Tae Ryom Oh, Su Hyun Song, Hong Sang Choi, Chang Seong Kim, Seung Hyeok Han, Kyung Pyo Kang, Young Joo Kwon, Soo Wan Kim, Seong Kwon Ma, Eun Hui Bae

**Affiliations:** 1 Department of Internal Medicine, Chonnan National University Medical School & Hospital, Gwangju 61469, Korea; tryeomoh@daum.net (T.R.O.); sudang_@naver.com (S.H.S.); hongsang38@hanmail.net (H.S.C.); laminion@hanmail.net (C.S.K.); kdksw@hanmail.net (S.W.K.); 2 Department of Internal Medicine, College of Medicine, Institute of Kidney Disease Research, Yonsei University, Seoul 03722, Korea; hansh@yuhs.ac; 3 Department of Internal Medicine, Research Institute of Clinical Medicine, Jeonbuk National University Medical School, Jeonju 54907, Korea; kpkang@jbnu.ac.kr; 4 Department of Internal Medicine, Korea University College of Medicine, Seoul 02841, Korea; yjkwon@korea.ac.kr

**Keywords:** glomerulonephritis, IgA nephropathy, hemoglobin, anemia, risk factors, prognosis, disease progression

## Abstract

Immunoglobin A (IgA) nephropathy causes chronic kidney disease worldwide. Therefore, identifying risk factors associated with the progression of IgA nephropathy is crucial. Anemia is a common complication of chronic kidney disease; however, few studies have investigated the effect of serum hemoglobin on the renal prognosis of IgA nephropathy. This study aimed to determine the effect of serum hemoglobin on the progression of IgA nephropathy. We retrospectively analyzed 4326 patients with biopsy-proven IgA nephropathy. We evaluated the effect of serum hemoglobin on IgA nephropathy progression using Kaplan–Meier survival analyses, the log-rank test, and the Cox proportional hazards model. The primary end-point was progression of IgA nephropathy, defined as dialysis initiation or kidney transplantation. Serum hemoglobin showed a nonlinear relationship with the progression of IgA nephropathy. The Cox proportional hazards model showed that the risk of progression of IgA nephropathy decreased 0.87 times for every 1.0 g/dL increase in serum hemoglobin. In subgroup analyses, reduced serum hemoglobin was an independent risk factor for IgA nephropathy progression only in women. There was no statistically significant interaction of serum hemoglobin between men and women (*P*_interaction_ = 0.177). Results of Sensitivity analysis were robust and consistent. Serum hemoglobin at diagnosis was an independent predictor for IgA nephropathy progression.

## 1. Introduction

In Korea, chronic glomerulonephritis (GN) is the third most common cause of end-stage renal disease (ESRD) [1]. Immunoglobulin A (IgA) nephropathy is the most common type of GN in Korea and also worldwide [2,3,4]. Among biopsy-proven GN, the prevalence of IgA nephropathy is also steadily increasing in Korea [3,5]. If GN, including IgA nephropathy, is not properly managed it eventually proceeds to ESRD, which causes a large socioeconomic burden [6]. The 30-year renal survival rate for IgA nephropathy is poor, at approximately 50.3% [7]. Due to the poor renal prognosis of IgA nephropathy, identification of modifiable risk factors for the progression of IgA nephropathy is important. Traditional risk factors of IgA nephropathy include decreased renal function, hypertension, and proteinuria at diagnosis [8]. Additionally, genetic factors [9], hyperuricemia [10,11], dyslipidemia [11], and smoking [12] have been shown to be related to progression of IgA nephropathy.

Anemia is one of the most frequent complications of chronic kidney disease (CKD), and the prevalence of anemia in CKD patients has been reported as ranging from 8.4% at stage 1 to 53.4% at stage 5 [13]. CKD and anemia are known to be related through various mechanisms, and recent studies show that anemia might lead to hypoxic injury in end-organs, including the kidneys, through the hypoxia-inducible factor (HIF) signaling pathway [14,15]. The HIF pathway is thought to be associated with disease progression, and related research has been conducted in CKD patients [16,17]. IgA nephropathy is a major cause of CKD. In IgA nephropathy, anemia, which is related to the HIF pathway, might have a significant correlation with renal prognosis [18]. However, the association between serum hemoglobin and the progression of IgA nephropathy has not been sufficiently studied [19,20]. In addition, there are no large-scale studies that have investigated the effect of serum hemoglobin at the time of diagnosis of IgA nephropathy on renal prognosis. Therefore, the purpose of this study was to identify the association between serum hemoglobin at diagnosis and the prognosis of IgA nephropathy.

## 2. Experimental Section

### 2.1. Data Source and Study Population

**Figure 1:**
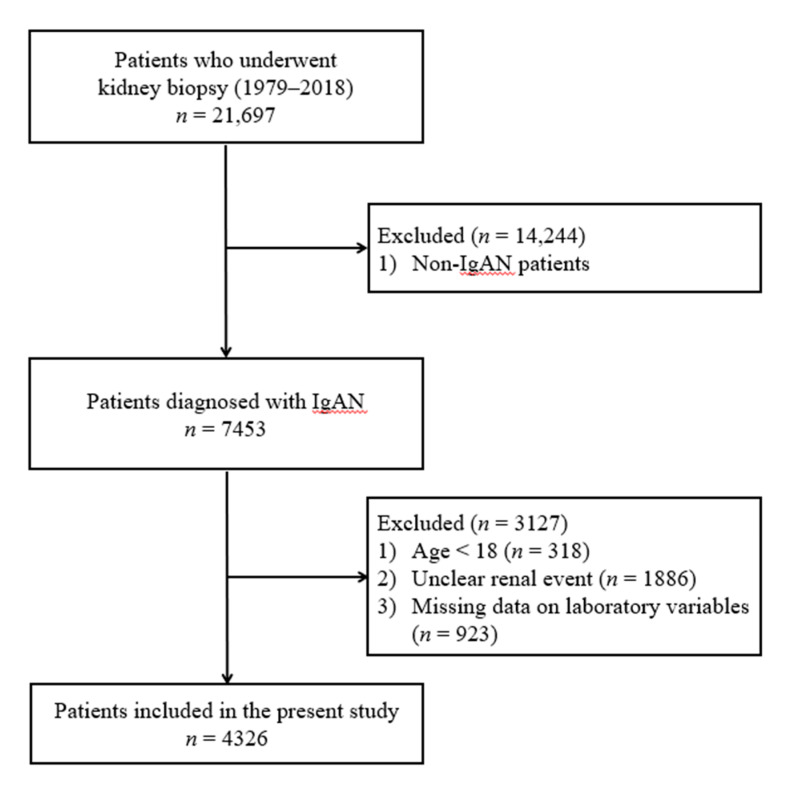
Enrollment of patients into this study.

From January 1979 to October 2018, 21,697 people received a kidney biopsy at 18 university hospitals in Korea, including Chonnam National University; 7453 of these patients were diagnosed with IgA nephropathy. The 18 hospitals were described in a previously published paper [10]. Patients were excluded for the following reasons: under 18 years of age (*n* = 318), did not know whether the renal event occurred (*n* = 1886), missing data (*n* = 923) on laboratory variables including serum hemoglobin. Finally, a total of 4326 patients were analyzed in this study (Figure 1).

### 2.2. End Point, Definitions, and Measurement

The primary end point of this study was the progression of IgA nephropathy, which was defined as a composite renal outcome consisting of a 2-fold increase in baseline serum creatinine or a 50% reduction of eGFR or the initiation of dialysis or kidney transplantation during the follow-up period. All laboratory tests, including serum hemoglobin level, were performed at the time of taking renal biopsy. Anemia was defined as hemoglobin levels below 13 g/dL in men and below 12 g/dL in women [21]. Smoking history was divided into current smoker, ex-smoker, and nonsmoker. For statistical analysis, we performed log transformation of urine protein creatinine ratio, which showed a skewed distribution. The estimated glomerular filtration rate (eGFR) was calculated using the original Modification of Diet in Renal Disease equation for adults and the height-independent equation for children [22].

### 2.3. Statistical Analysis

For continuous variables, data with normal distributions were described as means with standard deviations, and skewed data were expressed as medians with interquartile range (IQR). We used the Shapiro-Wilk test to determine normality. We used the Student’s t-test for normally distributed data and the Mann-Whitney U test for skewed data to identify differences and to compare laboratory findings and clinical characteristics between the groups. We expressed categorical variables as number of participants (percentage). We used chi-squared test to compare the two groups and the Cochran–Armitage trend test to compare more than two categories. The Kaplan–Meier survival curve with the log-rank test and the univariate Cox proportional hazards model were used to evaluate the association between serum hemoglobin and the progression of IgA nephropathy. We applied the multivariate Cox proportional hazards model to adjust for variables that may affect the progression of IgA nephropathy. We analyzed the mutual influence between variables using the collinearity test. Proportional hazard assumption of the Cox proportional hazards model was verified by Schoenfeld residuals and log minus log survival plot. We measured time stratified effect of fixed baseline eGFR that violated the proportional hazard assumption. We split the entire follow-up duration into 24-month intervals.

Hazard ratio (HR) and 95% confidence interval (CI) were calculated to compare the risk of progression of IgA nephropathy. We visualized the relationship between serum hemoglobin and the progression of IgA nephropathy in both sexes with a restricted cubic spline curve. Data were analyzed and plotted using R language (version 4.0.2; The R Foundation for Statistical Computing, Vienna, Austria) [23]. All statistical tests were two-tailed, and *p*-values < 0.05 were considered statistically significant.

### 2.4. Ethics Approval and Consent to Participate

This study adhered to the tenets of the Declaration of Helsinki. As the database used in this study did not include personal identifiers and the study was retrospective and observational in design, the need for informed consent was waived. Ethical approval was received from the Chonnam National University Hospital Institutional Review Board (CNUH-EXP-2020-285).

## 3. Results

### 3.1. Clinical Characteristics of the Study Population

Data from a total of 4326 patients were analyzed, and the percentages of missing data in all variables were less than 10%, except for C-reactive protein (19.6%) and urine protein/creatinine ratio (18.2%). The clinical characteristics of the patients are summarized in Table 1. The mean age and median eGFR of the patients were 39.3 years and 75.0 mL/min/1.73 m^2^. The sex ratio of the study population was similar (men, 49.5%; women, 50.5%) and the prevalence of diabetes mellitus was 7.6% (329 of the 4326 patients). The median (IQR) follow-up duration in the study population was 73 (range, 32.7–117.7) months.

**Figure 2:**
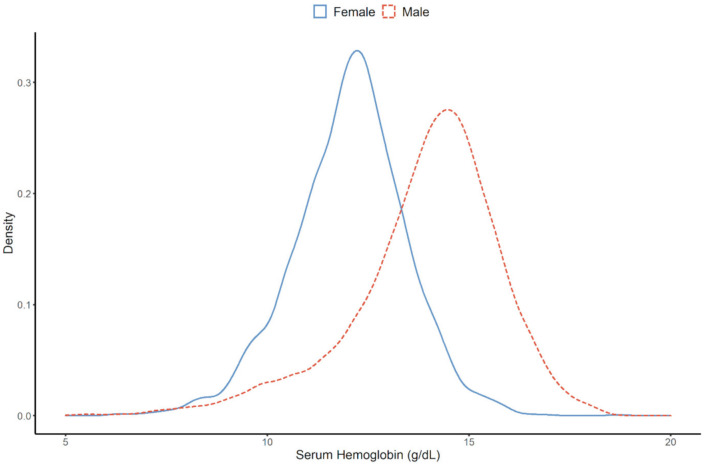
Difference in distribution of serum hemoglobin by sex.

We divided the patients into two groups based on the presence (anemia group) or absence (control group) of anemia. The mean (± SD) hemoglobin level was 14.0 ± 1.3 g/dL in the control group and 11.0 ± 1.2 g/dL in the anemia group. The distribution of hemoglobin by sex is shown in Figure 2. Statistically significant differences in many epidemiologic and clinical features were observed in the two groups, except in systolic and diastolic blood pressure, serum uric acid, and total cholesterol. The anemia group was older and had a higher proportion of women than the control group. The anemia group also had a higher prevalence of diabetes mellitus, and lower serum albumin levels and eGFR than the control group. The difference in UPCR (urine protein creatinine ratio) in both groups was statistically significant (*p* = 0.000). The median UPCR in the control group was 0.9 g/g Cr, and the median UPCR in the anemia group was 1.3 g/g Cr.

We also compared serum hemoglobin levels according to the CKD stages to investigate if the reduced hemoglobin simply means the result of worsening kidney function. Mean hemoglobin levels according to CKD stages were 13.5 g/dL in stage 1, 13.2 g/dL in stage 2, and 12.9 g/dL in stage 3a. Although differences of serum hemoglobin levels according to CKD stages were observed, we inferred that these degree of differences in serum hemoglobin will hardly have clinically significance. The detailed information is summarized in Supplementary Table S1.

### 3.2. Serum Hemoglobin, Anemia, and Progression of IgA Nephropathy

**Figure 3:**
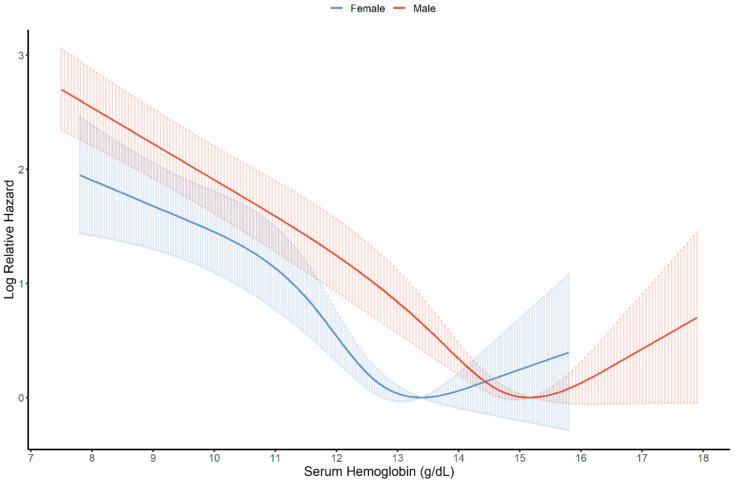
Restricted cubic spline curve of hazard ratio of serum hemoglobin for Immunoglobulin A (IgA) nephropathy progression by sex.

**Figure 4:**
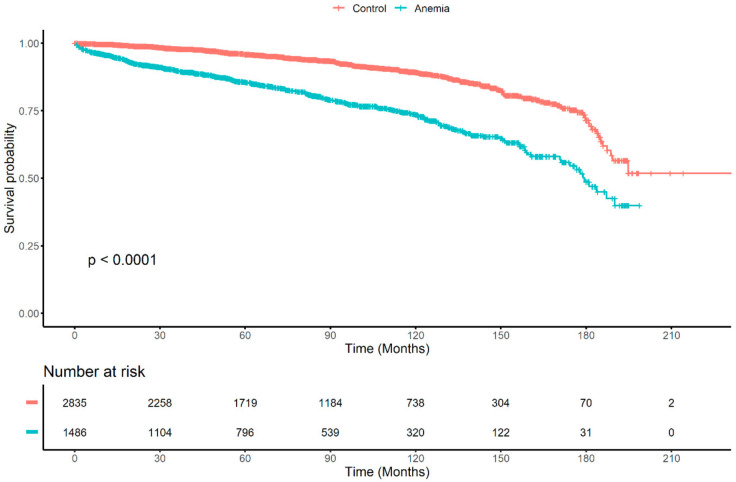
Kaplan–Meier survival analysis between anemia and control groups.

We visualized the nonlinear association between serum hemoglobin and the progression of IgA nephropathy in men and women using restricted cubic spline curves (Figure 3). The serum hemoglobin levels with the lowest log relative hazards were 15.182 g/dL in men and 13.388 g/dL in women. We analyzed the effect of serum hemoglobin and anemia on the progression of IgA nephropathy using Cox proportional hazards models (Table 2). We found that an increase of 1 g/dL in serum hemoglobin lowered the risk of progression of IgA nephropathy by 0.87 times in all patients. In the Kaplan–Meier survival analysis, the anemia group had a poor prognosis compared to the control group (Figure 4). Anemia was an independent risk factor for the progression of IgA nephropathy in the entire study population and female. Cox proportional hazard models showed that reduced serum hemoglobin significantly correlated with the risk of IgA nephropathy. In the fully adjusted model (model 3), the HRs (CIs) of anemia were 1.347 (1.016;1.787) in all patients, 1.190 (0.812;1.743) in men, and 1.674 (1.097;2.554) in women. There was no statistically significant interaction of serum hemoglobin between men and women (*p* for interaction = 0.177). We summarized the final Cox proportional models in Supplementary Table S2.

### 3.3. Sensitivity Analyses

We performed sensitivity analyses to prove the robustness of the results of this study. We divided the serum hemoglobin levels into seven categories (<10, 10–11, 11–12, 12–13, 13–14, 14–15, and >15 g/dL). The clinical characteristics of the seven categories are summarized in Supplementary Table S3. The clinical characteristics were analyzed using Kaplan–Meier survival analysis with the log-rank test and Cox proportional hazards models. In the Kaplan–Meier survival analysis, the <10 g/dL category showed the lowest survival probability and the other categories had similar survival probability (Supplementary Figure S1). The < 10 category was statistically significant in the fully adjusted model (model 3); however, the other categories were not statistically significant in these analyses (Table 3). Compared to the reference category (12–13 g/dL), there was an approximately 1.84-fold increase in the risk of IgA progression in the <10 g/dL category.

## 4. Discussion

In this study, which analyzed 4326 patients with IgA nephropathy, we found that serum hemoglobin level at diagnosis was an independent risk factor of disease progression. In addition, there was no sex-specific effect of serum hemoglobin on the progression of IgA nephropathy.

Renal anemia is due to various mechanisms, including decreased erythrocyte survival [24,25], erythropoietin (EPO) deficiency [26], inhibition of erythropoiesis [27], and imbalances in iron homeostasis [28,29]. Among these mechanisms, EPO deficiency and functional iron deficiency are associated with the HIF signaling pathway and have recently begun receiving attention [30]. HIF is a major factor that mediates the physiological response to hypoxia [30], maintains oxygen balance, and protects against cellular damage [31]. HIF is a heterodimer composed of alpha and beta subunits, and HIF-prolyl hydroxylase (PH) enzymes require oxygen in order to regulate HIF. In hypoxic conditions, HIF-PH enzyme functions decrease, which in turn stabilizes HIF [32,33]. Stabilization of HIF upregulates EPO and other genes [34].

Anemia in CKD is induced by hypoxic injury of the kidney due to decreased oxygen delivery and aggravated organ damage [35]. Previous studies have indicated an association between anemia-induced hypoxia and glomerular disease through the HIF-1α signaling pathway. This pathway is related to podocyte injury and promotion of proteinuria [18,36]. In addition, the relationship between anemia and pathological changes in renal tubulointerstitium has been reported [37,38]. Anemia-induced hypoxic injury on the glomerulus and the tubulointerstitium might explain the association of anemia with poor renal prognosis in patients with IgA nephropathy. In this study, the anemia group showed increased proteinuria compared to the control group, which is consistent with the results of previous studies [18,36]. Increased proteinuria might be associated with the HIF-1α signaling pathway. In the RENAAL study, the relationship between anemia and rapid decline of renal function was also reported [39], and results of that study are consistent with those of the present study.

In this study, we analyzed the effect of decreased serum hemoglobin on progression of IgA nephropathy. It is known that serum hemoglobin level decreases depending on the CKD stage. However, we utilized the hemoglobin level at the time of renal biopsy, and most of the patients were CKD stage 3 or lower (3593 of the 4326 patients). As shown in supplementary Table S1, it was difficult to evaluate that the difference in hemoglobin levels according to CKD stages had clinical significance. Although there was a statistical difference in serum hemoglobin according to the CKD stage, it was assumed that this difference was due to the large sample size. Based on these points, we evaluated serum hemoglobin as an independent risk factor for IgA nephropathy. Serum hemoglobin levels, which had the lowest log relative hazard, differed by sex. This phenomenon might be related to the difference in natural hemoglobin levels of both sexes [40]. In healthy men and women, an approximately 12% difference of mean hemoglobin levels in venous blood has been reported, with men having higher mean hemoglobin levels than women [41]. This might be caused by a stimulatory effect of androgen in men on erythropoiesis in bone marrow and in the kidneys, and the inhibitory effect of estrogen on bone marrow in women [42,43]. However, despite having different venous hemoglobin levels, both sexes maintain similar microcirculatory hematocrit levels using various mechanisms, including the Fahraeus effect, and also maintain similar levels of tissue oxygenation [40]. Although, in the subgroup analysis, serum hemoglobin did not show statistical significance for IgA progression in men, there was no interaction effect between serum hemoglobin and sex on the progression of IgA nephropathy in this study. Considering the absence of an interaction between serum hemoglobin and sex on the progression of IgA nephropathy, we presumed that there was no difference in the mechanism of renal injury between men and women.

Our data have many strengths, including a large study population, long follow-up period, and consistent and robust results. Despite these strengths, this study had some limitations. First, because of the study’s observational study, we could not evaluate the causal relationship between serum hemoglobin and the progression of IgA nephropathy. However, observational studies are nonetheless valuable tools for evaluation of epidemiological association, and we capitalized on complimentary analytic methods to effectively assess the association between serum hemoglobin and the progression of IgA nephropathy [44]. Second, we could not analyze the histopathological types of IgA nephropathy because information regarding types of IgA nephropathy were not available. Third, due to data limitations, we could not evaluate the effect of iron status and usage of drugs, such as immunosuppressant, renin–angiotensin system inhibitors, and EPO. Fourth, we could not evaluate the variability of serum hemoglobin. As kidney disease progresses, the serum hemoglobin level does not remain constant, but rather exhibits variability. In this study, we could not analyze the effects of the variability of serum hemoglobin level due to limitation of data availability. Finally, we could not eliminate all sources of bias and confounding factors.

## 5. Conclusions

Serum hemoglobin at diagnosis was an independent risk factor for the progression of IgA nephropathy.

## Figures and Tables

**Table 1:** Clinical characteristics of study population.

Characteristics	Missing Data (*n* (%))	All Subjects (*n* = 4326)	Control (*n* = 2839)	Anemia (*n* = 1488)	*p*-Value
Age (year)	0 (0)	39.3 ± 14.1	37.1 ± 13.6	43.6 ± 14.0	<0.001
Male (%)	0 (0)	2141 (49.5)	1644 (57.9)	497(33.4)	<0.001
Height (cm)	339 (7.8)	164.9 [158.0;172.0]	167.0 [160.0;173.5]	160.5 [156.0;167.0]	<0.001
Weight (kg)	192 (4.4)	63.6 [55.8;72.5]	66.0 [58.0;75.0]	59.0 [52.9;67.0]	<0.001
Body mass index	353 (8.2)	23.4 [21.1;26.0]	23.8 [21.5;26.4]	22.8 [20.5;25.0]	<0.001
Diabetes mellitus (%)	5 (0.1)	329 (7.6)	174 (6.1)	155 (10.4)	<0.001
Systolic blood pressure (mmHg)	239 (5.5)	123.9 ± 16.5	123.7 ± 15.9	124.3 ± 17.5	0.349
Diastolic blood pressure (mmHg)	240 (5.5)	123.9 ± 16.5	77.5 ± 11.4	77.3 ± 11.8	0.698
Smoking history (%)	388 (8.97)				0.000
Never		3109 (78.9)	1993 (77.0)	1116 (82.7)	
Ex		333 (8.5)	223 (8.6)	110 (8.2)	
Current		496 (12.6)	373 (14.4)	123 (9.1)	
Serum uric acid (mg/dL)	0 (0)	6.0 ± 1.8	6.0 ± 1.7	6.1 ± 1.9	0.789
Hemoglobin (g/dL)	0 (0)	13.0 ± 1.9	14.0 ± 1.3	11.0 ± 1.2	<0.001
Serum albumin (mg/dL)	16 (0.4)	3.9 [3.6;4.2]	4.0 [ 3.7;4.3]	3.7 [ 3.3;4.0]	<0.001
Creatinine (mg/dL)	5 (0.1)	1.0 [0.8;1.3]	1.0 [ 0.8;1.2]	1.1 [ 0.8;1.7]	<0.001
eGFR (ml/min/1.73 m^2^)	5 (0.1)	75.0 [54.4;95.8]	80.7 [63.2;100.1]	60.9 [36.8;84.2]	<0.001
CKD stages (%)	5 (0.1)				<0.001
1		1338 (30.9)	1048 (36.9)	290 (19.5)	
2		1645 (38.0)	1178 (41.5)	467 (31.4)	
3a		610 (14.1)	374 (13.2)	236 (15.9)	
3b		431 (10.0)	183 (6.4)	248 (16.7)	
4		231 (5.3)	45 (1.6)	186 (12.5)	
5		66 (1.5)	7 (0.2)	59 (4.0)	
Total cholesterol (mg/dL)	240 (5.5)	184.0 [157.0;215.0]	184.0 [157.0;215.0]	184.0 [157.0;215.0]	0.623
C-reactive protein (mg/dL)	848 (19.6)	0.2 [0.1;0.6]	0.2 [ 0.0;0.6]	0.2 [ 0.1;0.8]	<0.001
Urine protein creatinine ratio	788 (18.2)	1.0 [0.5;2.1]	0.9 [ 0.4;1.8]	1.3 [ 0.6;2.8]	<0.001
Follow-up duration (months)	0 (0)	73.0 [32.7;117.7]	78.1 [34.9;120.8]	63.8 [28.3;111.2]	<0.001

Abbreviation: eGFR, estimated Glomerular Filtration Rate; CKD, chronic kidney disease.

**Table 2:** Hazard ratio of hemoglobin for renal prognosis of IgA nephropathy with Cox proportional hazard models by sex.

	Total Subjects		Male		Female	
	HR [95% CI]	*p*-Value	HR [95% CI]	*p*-Value	HR [95% CI]	*p*-Value
*Serum hemoglobin* (g/dL)						
Crude	0.768 [0.736;0.802]	<0.001	0.719 [0.687;0.752]	<0.001	0.701 [0.645;0.763]	<0.001
Model 1	0.765 [0.700;0.835]	<0.001	0.786 [0.741;0.835]	<0.001	0.771 [0.704;0.844]	<0.001
Model 2	0.875 [0.799;0.958]	0.004	0.916 [0.857;0.978]	0.008	0.892 [0.8097;0.983]	0.022
Model 3	0.871 [0.773;0.983]	0.025	0.937 [0.858;1.023]	0.145	0.875 [0.768;0.998]	0.046
					*p* for interaction = 0.177	
*Presence of anemia*						
Crude	2.780 [2.353;3.285]	<0.001	3.688 [2.964;4.590]	<0.001	2.770 [2.112;3.633]	<0.001
Model 1	2.267 [1.698;3.025]	<0.001	2.433 [1.855;3.192]	<0.001	2.203 [1.645;2.951]	<0.001
Model 2	1.757 [1.287;2.398]	<0.001	1.306 [0.964;1.770]	0.085	1.714 [1.243;2.364]	0.001
Model 3	1.675 [1.112;2.522]	0.002	1.241 [0.871;1.768]	0.232	1.674 [1.097;2.554]	0.017
			*p* for interaction = 0.226			

Model 1 (crude + age, sex (exclude sex in subgroup analysis), diabetes mellitus, systolic blood pressure) was stratified with hypoalbuminemia; Model 2, crude + age, sex (exclude sex in subgroup analysis), diabetes mellitus, serum albumin, systolic blood pressure, smoking history, uric acid and time stratified estimated glomerular filtration rate; Model 3, Model 2 + log(urine protein creatinine ratio), total cholesterol, C- reactive protein; Abbreviation: CI, confidence interval; HR, hazard ratio; IgA, immunoglobulin A.

**Table 3:** Hazard ratio of categories of hemoglobin for renal prognosis of IgA nephropathy with Cox proportional hazard models.

	Categories of Hemoglobin (g/dL)						
	<10 (*n* = 254)	10–11 (*n* = 344)	11–12 (*n* = 663)	12–13 (*n* = 886)	13–14 (*n* = 784)	14–15 (*n* = 736)	>15 (*n* = 659)
Crude	4.577 [3.423;6.120]	2.382 [1.749;3.244]	1.693 [1.272;2.255]	Reference	0.954 [0.700;1.298]	0.820 [0.593;1.133]	0.910 [0.656;1.264]
Model 1	3.005 [2.169;4.163]	1.927 [1.374;2.702]	1.677 [1.232;2.282]	Reference	0.808 [0.577;1.132]	0.638 [0.445;0.915]	0.619 [0.427;0.898]
Model 2	1.530 [1.074;2.178]	1.291 [0.900;1.853]	1.459 [1.058;2.013]	Reference	1.059 [0.747;1.502]	0.961 [0.664;1.391]	0.987 [0.672;1.451]
Model 3	1.837 [1.189;2.837]	1.027 [0.638;1.655]	1.205 [0.793;1.831]	Reference	1.132 [0.732;1.750]	1.029 [0.643;1.646]	1.214 [0.750;1.967]

Model 1 (crude + age, sex (exclude sex in subgroup analysis), diabetes mellitus, systolic blood pressure) was stratified with hypoalbuminemia; Model 2, crude + age, sex (exclude sex in subgroup analysis), diabetes mellitus, serum albumin, systolic blood pressure, smoking history, uric acid and time stratified estimated glomerular filtration rate; Model 3, Model 2 + log(urine protein creatinine ratio), total cholesterol, C- reactive protein; Abbreviation; IgA, immunoglobulin A.

## Data Availability

The data presented in this study are available with permission from the KoGNET Academic Committee.

## Supplementary Materials

The following are available online at https://www.mdpi.com/2077-0383/10/2/363/s1, Table S1. Hemoglobin levels according to stages of chronic kidney disease; Table S2. Final Cox proportional models with time stratified effects of eGFR; Table S3. Clinical characteristics of study population stratified with categories of hemoglobin; Figure S1. Kaplan Meier survival analysis among seven categories of hemoglobin.
