# Supplementary material for: The Association between Serum Hemoglobin and Renal Prognosis of IgA Nephropathy
Source: J Clin Med. 2021 Jan 19;10(2):363. doi: 10.3390/jcm10020363 (PMC7835832; doi:10.3390/jcm10020363)
Supplement: Supplementary file 1 [file jcm-10-00363-s001.pdf]

Table S1. Hemoglobin levels according to stages of chronic kidney disease.

	**CKD stage 1 (n= 1252)**	**CKD stage 2 (n= 1552)**	**CKD stage 3a (n=570)**	***p*-value**
Serum hemoglobin (g/dl)	13.5 ± 1.7	13.2 ± 1.7	12.9 ± 1.8	<0.001

Table S2. Final Cox proportional models with time stratified effects of eGFR.

	**Total subjects**		**Male**		**Female**	
	HR [95% CI]	*p*-value	HR [95% CI]	*p*-value	HR [95% CI]	*p*-value
Serum hemoglobin (g/dL)						
Age	0.998 [0.989;1.006]	0.575	1.001 [0.989;1.013]	0.878	0.993 [0.979;1.007]	0.324
Hemoglobin	0.872 [0.777; 0.979]	0.025	0.936 [0.863;1.015]	0.111	0.875 [0.768;0.998]	0.046
Male	0.660 [0.144;3.031]	0.593	-	-		
Diabetes mellitus	1.018 [0.685;1.514]	0.929	1.018 [0.615;1.686]	0.944	1.003 [0.510;1.969]	0.994
Systolic blood pressure	0.999 [0.994;1.006]	0.927	1.001 [0.994;1.008]	0.743	0.998 [0.987;1.009]	0.671
Serum albumin	0.816 [0.643;1.036]	0.095	0.831 [0.616;1.121]	0.226	0.827 [0.542;1.262]	0.379
Serum uric acid	1.025 [0.956;1.101]	0.486	1.011 [0.928;1.101]	0.805	1.096 [0.951;1.263]	0.207
Log(UPCR)	1.350 [1.173;1.555]	< 0.001	1.340 [1.114;1.612]	0.002	1.370 [1.090;1.724]	0.007
Total cholesterol	0.998 [0.996;1.000]	0.056	0.999 [0.996;1.002]	0.381	0.997 [0.994;1.001]	0.105
Smoking history						
Ex	0.723 [0.476;1.098]	0.128	0.732 [0.472;1.134]	0.163	0.389 [0.053;2.860]	0.354
Current	0.896 [0.638;1.259]	0.527	0.889 [0.611;1.294]	0.540	0.948 [0.397;2.260]	0.903
C-reactive protein	0.952 [0.907;0.999]	0.045	0.952 [0.896;1.011]	0.110	0.955 [0.883;1.034]	0.255
eGFR:<24 month	0.936 [0.923;0.949]	< 0.001	0.939 [0.923;0.955]	< 0.001	0.933 [0.913;0.954]	< 0.001
eGFR:24-48 months	0.966 [0.954;0.979]	< 0.001	0.967 [0.952;0.983]	< 0.001	0.968 [0.946;0.990]	0.004
eGFR:48-72 months	0.972 [0.959;0.985]	< 0.001	0.976 [0.960;0.993]	0.005	0.966 [0.944;0.989]	0.003
eGFR:72-96 months	0.976 [0.964;0.987]	< 0.001	0.973 [0.958;0.989]	< 0.001	0.981 [0.963;1.000]	0.050
eGFR:96-120 months	0.964 [0.948;0.981]	< 0.001	0.971 [0.951;0.991]	0.005	0.953 [0.924;0.984]	0.003
eGFR:120-144 months	0.964 [0.949;0.980]	< 0.001	0.965 [0.943;0.987]	0.002	0.967 [0.945;0.990]	0.006
eGFR:>144 months	1.004 [0.987;1.022]	< 0.001	1.005 [0.983;1.028]	0.646	1.005 [0.976;1.035]	0.737
Presence of anemia						
Age	0.998 [0.989;1.006]	0.581	1.001 [0.990;1.013]	0.811	0.992 [0.978;1.006]	0.264
Anemia	1.675 [1.112;2.522]	0.014	1.190 [0.812;1.743]	0.372	1.674 [1.097;2.554]	0.017
Male	1.960 [1.286;2.988]	0.002	-			
Diabetes mellitus	1.025 [0.689;1.524]	0.904	1.036 [0.626;1.716]	0.890	1.003 [0.510;1.970]	0.994
Systolic blood pressure	1.000 [0.994;1.006]	0.989	1.001 [0.994;1.008]	0.761	0.999 [0.988;1.009]	0.778
Serum albumin	0.795 [0.628;1.008]	0.058	0.816 [0.606;1.099]	0.180	0.786 [0.517;1.196]	0.261

Serum uric acid	1.028 [0.958;1.103]	0.449	1.015 [0.932;1.105]	0.736	1.095 [0.950;1.262]	0.210
Log(UPCR)	1.353 [1.175;1.557]	< 0.001	1.350 [1.122;1.623]	0.001	1.364 [1.086;1.714]	0.007
Total cholesterol	0.998 [0.996;1.000]	0.040	0.998 [0.996;1.001]	0.274	0.997 [0.994;1.001]	0.099
Smoking history						
Ex	0.713 [0.469;1.083]	0.113	0.717 [0.463;1.110]	0.135	0.391 [0.053;2.884]	0.357
Current	0.869 [0.619;1.220]	0.416	0.864 [0.595;1.255]	0.443	0.858 [0.360;2.045]	0.730
C-reactive protein	0.953 [0.908;0.999]	0.046	0.950 [0.895;1.009]	0.094	0.958 [0.887;1.035]	0.280
eGFR:<24 month	0.934 [0.921;0.946]	< 0.001	0.936 [0.920;0.952]	< 0.001	0.932 [0.911;0.953]	< 0.001
eGFR:24-48 months	0.966 [0.953;0.979]	< 0.001	0.966 [0.950;0.982]	< 0.001	0.968 [0.946;0.990]	0.005
eGFR:48-72 months	0.971 [0.958;0.984]	< 0.001	0.975 [0.959;0.992]	0.003	0.966 [0.944;0.989]	0.003
eGFR:72-96 months	0.975 [0.963;0.987]	< 0.001	0.972 [0.956;0.988]	< 0.001	0.981 [0.963;1.000]	0.054
eGFR:96-120 months	0.963 [0.947;0.980]	< 0.001	0.970 [0.950;0.990]	0.004	0.953 [0.924;0.984]	0.003
eGFR:120-144 months	0.964 [0.948;0.980]	< 0.001	0.964 [0.942;0.987]	0.002	0.968 [0.946;0.991]	0.006
eGFR:>144 months	1.004 [0.986;1.021]	< 0.001	1.004 [0.982;1.027]	0.719	1.004 [0.976;1.034]	0.769

Abbreviation: CI, confidence interval; eGFR, estimated Glomerular Filtration Rate; HR, hazard ratio; UPCR, urine protein creatinine ratio.

Table S3. Clinical characteristics of study population stratified with categories of hemoglobin.

**Characteristics**	**Missing data (n (%))**	**All subjects (n=4326)**	**Hemoglobin (g/dL)** <10 (n=254)	10-11 (n=344)	11-12 (n=663)	12-13 (n=886)	13-14 (n=784)	14-15 (n=736)	>15 (n=659)	***p* for trend**
Age (year)	0 (0)	39.3 ± 14.1	48.1 ± 15.7	44.7 ± 14.0	41.5 ± 12.7	40.8 ± 12.7	39.7 ± 13.5	35.2 ± 13.8	33.2 ± 13.8	< 0.001
Male (%)	0 (0)	2141 (49.5)	87 (34.3)	71 (20.6)	112 (16.9)	226 (25.5)	421 (53.7)	607 (82.5)	616 (93.5)	< 0.001
Height (cm)	339 (7.8)	164.9 [158.0;172.0]	160.0 [156.0;166.0]	159.0 [155.0;164.0]	159.8 [155.0;164.0]	160.3 [156.0;166.5]	166.0 [159.0;172.0]	170.9 [165.0;175.6]	172.5 [168.0;177.0]	< 0.001
Weight (kg)	192 (4.4)	63.6 [55.8;72.5]	57.8 [51.0;66.2]	57.8 [52.0;63.7]	57.0 [52.0;64.8]	59.6 [54.0;67.0]	65.0 [57.6;72.5]	69.0 [62.0;77.0]	73.0 [66.1;82.0]	< 0.001
Body mass index	353 (8.2)	23.4 [21.1;26.0]	22.7 [20.1;24.8]	22.7 [20.3;24.6]	22.3 [20.5;25.1]	22.9 [20.9;25.4]	23.5 [21.4;26.3]	23.9 [21.8;26.5]	24.8 [22.5;27.4]	< 0.001
Diabetes mellitus (%)	5 (0.1)	329 (7.6)	51 (20.1)	33 (9.6)	47 (7.1)	67 (7.6)	57 (7.3)	43 (5.8)	31 (4.7)	< 0.001
Systolic blood pressure (mmHg)	239 (5.5)	123.9 ± 16.5	127.4 ± 17.9	126.0 ± 17.6	121.8 ± 16.5	121.5 ± 16.9	122.9 ± 15.8	125.1 ± 15.1	126.7 ± 16.2	< 0.001
Diastolic blood pressure (mmHg)	240 (5.5)	123.9 ± 16.5	78.7 ± 12.1	78.3 ± 11.8	76.0 ± 11.5	76.2 ± 11.6	76.7 ± 11.3	78.3 ± 11.0	79.3 ± 11.8	< 0.001

Smoking history (%)	388 (8.97)									< 0.001
Never		3109 (78.9)	188 (82.8)	375 (63.2)	266 (86.4)	530 (87.3)	721 (87.5)	559 (77.9)	470 (71.1)	
Ex		333 (8.5)	19 (8.4)	66 (11.1)	18 (5.8%)	32 (5.3)	53 (6.4)	73 (10.2)	72 (10.9)	
Current		496 (12.6)	20 (8.8)	152 (25.6)	24 (7.8)	45 (7.4)	50 (6.1)	86 (12.0)	119 (18.0)	
Serum uric acid (mg/dl)	0 (0)	6.0 ± 1.8	6.7 ± 2.1	6.0 ± 1.9	5.5 ± 1.8	5.6 ± 1.8	5.9 ± 1.7	6.5 ± 1.6	6.7 ± 1.4	< 0.001
Hemoglobin (mg/dl)	0 (0)	13.0 ± 1.9	9.0 ± 0.9	10.5 ± 0.3	11.5 ± 0.3	12.4 ± 0.3	13.4 ± 0.3	14.4 ± 0.3	15.8 ± 0.7	< 0.001
Serum albumin (mg/dl)	16 (0.4)	3.9 [3.6; 4.2]	3.4 [2.9; 3.8]	3.6 [3.1; 3.9]	3.8 [3.5; 4.1]	3.9 [3.6; 4.2]	4.0 [3.7; 4.3]	4.1 [3.8; 4.4]	4.2 [3.9; 4.5]	< 0.001
Creatinine (mg/dl)	5 (0.1)	1.0 [0.8; 1.3]	1.6 [1.0; 2.8]	1.1 [0.8; 1.7]	0.9 [0.7; 1.3]	0.9 [0.7; 1.2]	1.0 [0.8; 1.2]	1.0 [0.9; 1.2]	1.0 [0.9; 1.2]	< 0.001
eGFR (ml/min/1.73m ^{2})	5 (0.1)	75.0 [54.4;95.8]	37.1 [20.8;58.8]	55.5 [32.8;81.6]	70.9 [49.8;89.2]	76.8 [56.1;97.8]	77.0 [60.4;95.0]	81.3 [62.8;101.3]	84.1 [65.8;104.1]	< 0.001
Total cholesterol (mg/dl)	240 (5.5)	184.0 [157.0;215.0]	182.5 [150.0;217.0]	189.0 [160.0;213.0]	184.0 [160.0;217.0]	186.0 [159.0;215.0]	185.0 [158.0;217.0]	178.5 [154.0;211.0]	185.0 [157.0;212.0]	0.134
C-reactive protein (mg/dl)	848 (19.6)	0.2 [0.1; 0.6]	0.3 [0.1; 1.7]	0.2 [0.0; 1.0]	0.2 [0.1; 0.6]	0.2 [0.0; 0.6]	0.2 [0.0; 0.5]	0.2 [0.1; 0.6]	0.2 [0.1; 0.5]	< 0.001
Urine protein creatinine ratio	788 (18.2)	1.0 [0.5; 2.1]	2.2 [0.8; 4.3]	1.6 [0.8; 3.6]	1.1 [0.6; 2.2]	1.0 [0.5; 1.9]	1.0 [0.5; 1.8]	0.8 [0.4; 1.6]	0.8 [0.3; 1.8]	< 0.001
Follow-up duration (months)	0 (0)	73.0 [32.7;117.7]	51.1 [14.6;100.3]	63.1 [26.0;113.5]	69.3 [32.7;114.2]	71.0 [34.6;116.0]	79.7 [34.3;120.1]	80.5 [35.9;123.8]	75.1 [34.8;120.9]	< 0.001

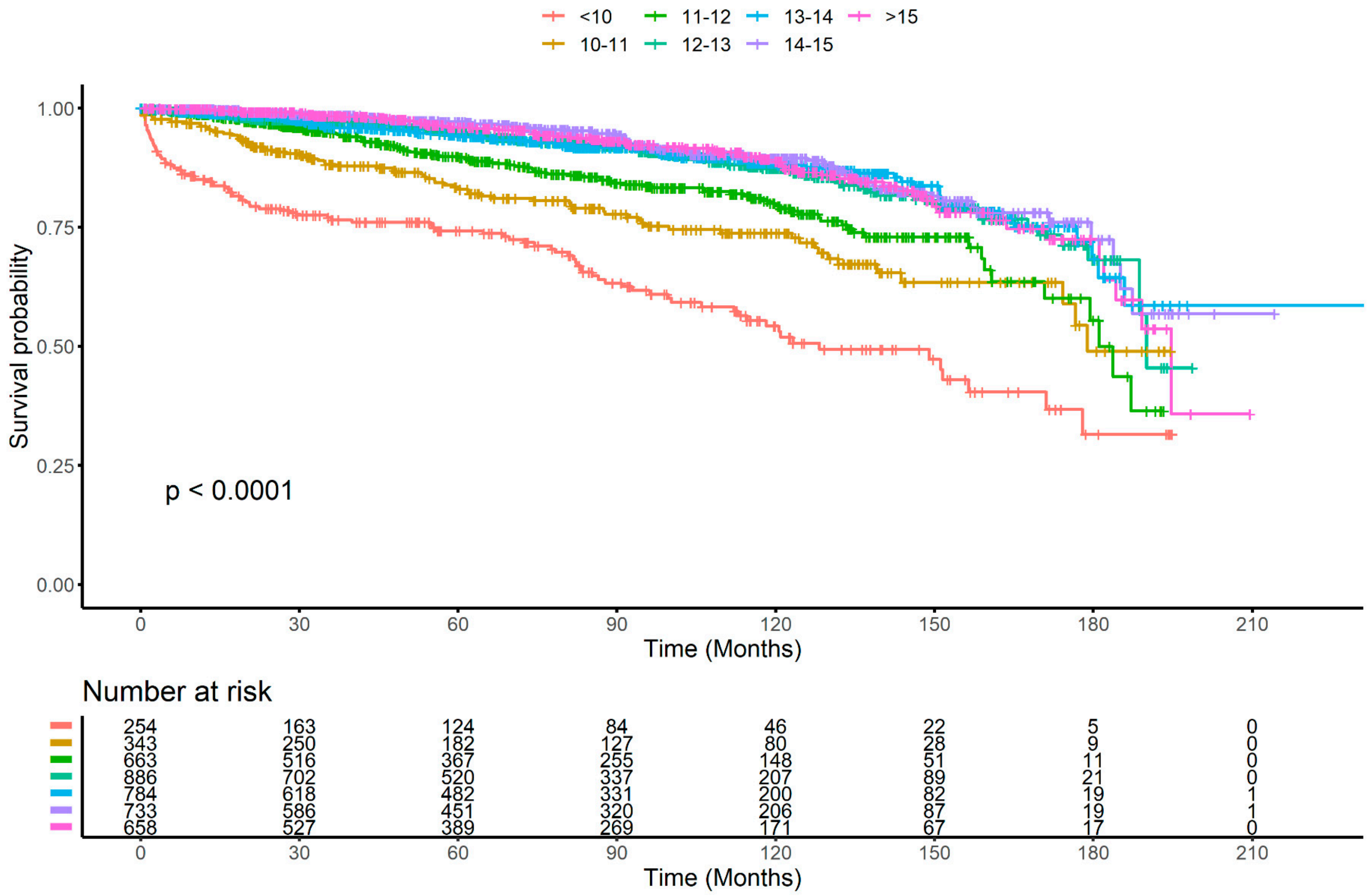

Figure S1. Kaplan Meier survival analysis among seven categories of hemoglobin.
